# Progress and Practice of Insulin Resistance in Pregnancy: From Pathogenesis to Management

**DOI:** 10.7150/ijms.136420

**Published:** 2026-09-03

**Authors:** Ya Zhu, Hui Gu, Ping Li

**Affiliations:** 1 Key Laboratory of Environmental Stress and Chronic Disease Control and Prevention, Ministry of Education (China Medical University), Shenyang, Liaoning, PR China; Environment and Non-Communicable Disease Research Center, Key Laboratory of Arsenic-related Biological Effects and Prevention and Treatment in Liaoning Province, School of Public Health, China Medical University, Shenyang, Liaoning, China.; 2 Department of Endocrinology, Shengjing Hospital of China Medical University, Shenyang 110022, China.; 3 NHC Key Laboratory of Congenital Malformation, Shengjing Hospital of China Medical University,110004, Shenyang, Liaoning, China.

**Keywords:** insulin resistance in pregnancy, gestational diabetes mellitus, obstetric outcomes, assessment, intervention

## Abstract

Evidence suggests that the degree of insulin resistance in pregnancy (IRiP) seems to affect pregnancy outcomes regardless of the presence of gestational diabetes. A physiological moderate increase in IRiP is a protective factor for fetal development, but excessive enhancement can lead to maternal metabolic disorders and placental dysfunction, increasing the risk of gestational diabetes and adverse pregnancy outcomes, as well as having adverse effects on the long-term health of the offspring. This review briefly explores the key factors influencing IRiP, including pregnancy-specific and non-pregnancy-specific factors. It also discusses the assessment methods for insulin resistance that can be implemented during pregnancy and the increased probabilities of adverse effects on both the mother and the offspring, such as preeclampsia or eclampsia, neonatal hypoglycemia, and the risk of obesity and metabolic diseases in the offspring in the future. Finally, the intervention strategies were briefly described, such as in terms of diet, exercise, and medication, etc. This provides a basis and insights for the management and research of IRiP.

## 1. Introduction

Gestational diabetes mellitus (GDM) is defined as abnormal glucose metabolism that occurs for the first time during pregnancy, the global prevalence rate is 10.5%-24.2%, and in China, it is approximately 11.9%^1^. GDM not only compromises maternal and fetal health, elevating risks of preeclampsia, macrosomia, and perinatal mortality, but also confers long-term metabolic disorder, significantly increasing the risk of type 2 diabetes, obesity, and cardiovascular disease in both mothers and their offspring^2^. GDM is a multifactorial disease, which may be influenced by the interaction of genetic, epigenetic and environmental factors, however, the exact cause is not yet fully understood^3^. Insulin resistance (IR) undergoes a physiological increase during pregnancy. When maternal insulin secretion fails to adequately compensate for IRiP, maternal glycemia rises progressively^4^. During normal pregnancy, the total number of β cells increases, within 10 days after the birth of the baby, the number of β cells returns to the normal level through mechanisms such as increasing β cell apoptosis, reducing proliferation, and reducing cell volume, but the balance between the growth and loss of β cells is strictly regulated, the normal number of functional β cells is usually able to compensate for the increase in IRiP, if this balance is improperly deviated from the normal induction of β cell growth and survival during the process of β cell expansion, the compensation by β cells may fail, and finally leading to GDM^5^.

IR is a pre-pathological state of many metabolic diseases, it refers to the state where insulin-targeted tissues, such as the liver, adipose tissue, and skeletal muscle, exhibit reduced responsiveness to physiological insulin levels^6^. Previously, people mainly focused on the impact of gestational hyperglycemia on the mother and fetus. However, recent studies have shown that, regardless of whether the pregnant women have GDM or not, excessive enhanced IRiP may increase the risk of adverse pregnancy outcomes^7^. Therefore, this review will independently of gestational hyperglycemia, systematically review the occurrence mechanism, assessment methods, and impact on obstetric outcomes of IRiP, aiming to identify the related public health issues of IRiP and provide intervention basis for clinical research.

## 2. The pathogenesis of insulin resistance in pregnancy

### 2.1 Pregnancy-specific factors

#### 2.1.1 Placental secretion of IR-promoting hormones

During the middle and late stages of pregnancy, the placenta secretes hormones in doses far exceeding the normal physiological levels to maintain normal physiological metabolism, including progesterone, cortisol, placental growth hormone, prolactin, IRiP involves complex interactions of hormones and cytokines produced by the placenta, adipose tissue, adrenal glands, ovaries, and immune cells, however, the exact causal relationship between specific hormones and IR has not been conclusively confirmed *in vivo*^8^-^10^. Current research indicates that most placental hormones have both positive and negative effects on IR, depending on factors such as hormone secretion levels, thus constituting an extremely complex regulatory role in IRiP^11^.

##### (1) Human placental growth hormone (hPGH)

hPGH is a polypeptide hormone that is continuously secreted by the placenta at an increasing concentration throughout pregnancy and participates in the growth of the fetus and placenta, fat decomposition and milk production^12^. During pregnancy, the content of growth hormone gradually increases with the increase of gestational weeks, in pregnant women with GDM, it regulates maternal metabolism through the growth hormone-insulin-insulin like growth factor 1 (IGF-1) axis, thereby affecting the growth of the fetus and the insulin sensitivity of the mother^13^. Furthermore, some scholars have found that hPGH in pregnant mice specifically increases the expression of p85α (the regulatory subunit of PI 3-kinase), thereby affecting the ability of insulin-stimulated p85-p110 heterodimers to bind to insulin receptor substrate 1 (IRS1) protein in skeletal muscle, reducing PI 3-kinase insulin signal transduction, and thus enhancing IRiP^14^, ^15^. Therefore, hPGH has been widely recognized as an important factor mediating IRiP.

##### (2) Human placental lactogen (hPL)

hPL is a polypeptide hormone, that can increase the concentration and activity of insulin in the circulation^12^. hPL induces lipolysis, increases free fatty acids in circulation, activates protein kinase C, and inhibits insulin signal transduction^16^. hPL may cause lipid metabolism disorders by interfering with the balance of lipoprotein lipase regulatory factors, and thereby promote the development of IRiP and GDM in a synergistic manner^17^. Meanwhile, the prolactin-like hormones secreted by the placenta, together with serum prolactin, activate the prolactin receptors on pancreatic β cells, promoting insulin secretion, this is a key adaptive response of the body to deal with the physiological IR during pregnancy^18^.

##### (3) Progestational hormone

Progestational hormone represents a class of sex hormones related to the changes in the uterus and vagina during the menstrual cycle, and is essential for the development of female secondary sexual characteristics and the maintenance of pregnancy^8^. High progestational hormone levels are associated with impaired glucose tolerance, exogenous progestational hormone can reduce insulin sensitivity, which subsequently leads to hyperglycemia^19^. Due to the effect of pregnancy hormones, IR is enhanced for most of the time during the second and third trimesters of pregnancy, by the 32nd week of pregnancy, progesterone reaches its peak, the increase in progesterone levels may further enhance IR by stimulating hepatic gluconeogenesis and impairing glucose uptake in peripheral tissues^8^, ^10^, ^20^.

##### (4) Estrogen

Estrogen is a steroid hormone, and in particular, estradiol (E2) has a rather complex effect on IRiP. On the one hand, it may protect the function of pancreatic β cells, reduce adipocyte hypertrophy and IR, and improve glucose utilization in the liver, indicating that estrogen has a weakening effect on IRiP; on the other hand, high concentrations of endogenous E2, especially during pregnancy, may reduce insulin sensitivity by decreasing the expression of glucose transporter 4 (GLUT-4) in skeletal muscle and interfere with the binding of insulin to insulin receptors, suggesting that excessive estrogen may lead to an enhanced IRiP^9^, ^11^.

#### 2.1.2 Abnormal lipids metabolism

Lipid metabolism is impaired by insufficient fatty acid oxidation, leading to lipid accumulation and redox imbalance, thereby promoting hepatic steatosis and IR^21^. High concentrations of free fatty acids and triglycerides inhibit the core pathways of insulin signal transduction in insulin target organs, impede glucose uptake and utilization, and trigger IR^22^. Pregnancy significantly alters lipid metabolism, and the inhibitory effect of glucose load on peripheral lipolysis weakens, leading to elevated levels of free fatty acids, cholesterol, triglycerides and ketone bodies. In pregnant women with GDM, these physiological changes are further amplified, showing more significant lipolysis resistance in the third trimester, which is due to the damage of the insulin signaling pathway in adipose tissue, this weakens the inhibitory effect of insulin on lipolysis, resulting in a significant increase in fasting free fatty acid concentration, which in turn activates hepatic gluconeogenesis, increases hepatic glucose output, and inhibits glucose uptake by skeletal muscle, further exacerbating IRiP^23^-^26^. Yu Bai *et al*. discovered that angiopoietin-like protein 8 (ANGPTL8), a secreted protein highly expressed in the liver and fat and involved in lipid metabolism, was highly expressed in the placenta of GDM mice, through a trophoblast cell model of IR induced by high insulin, they discovered that silencing of ANGPTL8 could reduce the IR level of these cells by inhibiting c-Jun N-terminal kinase (JNK) signal transduction^27^.

#### 2.1.3 Placenta-derived inflammatory factors

TNF-α is a pro-inflammatory cytokine and a key factor linking obesity, chronic inflammation and IR. During pregnancy, it is mainly synthesized and secreted by the placenta, and most of the TNF-α in the placenta is released into the maternal circulation during pregnancy^28^, ^29^. TNF-α is regarded as a central mediator of IR by interfering with the translocation of GLUT-4 to the cell membrane and through insulin signal transduction^30^. Placental TNF-α is a key inflammatory factor in IRiP, the level of placental TNF-α is significantly elevated in patients with GDM and is positively correlated with abnormal fasting blood glucose and glycated hemoglobin in GDM^29^. Studies have shown that IR during the late stage of pregnancy is significantly correlated with the changes of TNF-α in the placenta^31^. In addition, other inflammatory factors may also be related to IRiP, for instance, some studies have shown that in the placentas of patients with GDM, the phenomenon of pyroptosis is significantly increased, accompanied by the inflammatory markers associated with pyroptosis-interleukin-1β (IL-1β) and interleukin-18 (IL-18), particularly in patients with GDM, the levels of IL-1β and IL-18 in the placenta are closely related to the IR index^32^. Interleukin-6 (IL-6), Fatty Acid Binding Protein 4 (FABP4), and chemokines have also been studied for their role in exacerbating IR during pregnancy^10^.

#### 2.1.4 Multiparity

Parity refers to the number of live births a woman has in her lifetime and is a risk factor for the occurrence and recurrence of GDM^33^, ^34^. Studies have shown that multiparities may increase the level of IR, the overweight or obesity of the mother resulting from multiparities, the recurrent inflammatory responses, and the combined effect of these factors further exacerbate this relationship^35^. Multiparity is an independent predictor of obesity, approximately three quarters of women fail to regain their pre-pregnancy weight within one year after giving birth^36^, ^37^. In addition, regardless of whether obesity exists or not, during pregnancy, the mother's body increases the production of placental hormones, such as progesterone, estrogen, and placental lactogen, to adapt to the growing fetus, this further aggravates IR, the above effects especially have a cumulative impact on multiparous women^35^. However, some studies have indicated that parity is not directly related to insulin sensitivity or GDM, but is merely associated through the increase in maternal age and weight^34^. Therefore, whether there is a direct causal relationship between parity during pregnancy and IR still needs further exploration.

### 2.2 Non-pregnancy-specific factors

#### 2.2.1 Genetic Susceptibility

A variety of genome-wide association studies have shown that numerous genes are related to IR, due to the overlap in genetic background between GDM and type 2 diabetes, genes associated with type 2 diabetes may also be related to the risk of GDM, specifically. In terms of epidemiology, current literature has clearly reported single nucleotide polymorphisms (SNPs) related to IR during pregnancy and the functions of the genes they are located in, as shown in Table 1,^10^, ^38^.

#### 2.2.2 Pre-pregnancy of overweight or obesity

Obesity is a major health issue, overweight and obesity have become a global epidemic, the rising incidence of obesity among women of childbearing age will further increase the prevalence of obesity among pregnant women^39^. Pre-pregnancy overweight or obesity is a high-risk factor for GDM, hypertensive syndrome, and fetal growth disorders^40^. When GDM and pre-pregnancy overweight coexist, the risk of metabolic syndrome significantly increases^41^. Pre-pregnancy body mass index (BMI) is a factor influencing IR, and it has a certain correlation with insulin sensitivity during pregnancy, with a negative correlation between the two^42^. Women with a higher pre-pregnancy BMI (overweight or obese) have higher fasting insulin levels and a greater degree of IR in the early stage of pregnancy compared to those with a lower pre-pregnancy BMI, being overweight or obese during pregnancy may lead to earlier onset of IRiP in pregnant women^43^, ^44^. Compared with women with a normal BMI, those women with a higher pre-pregnancy BMI (overweight or obese) have a higher risk of excessive IRiP in the second trimester of pregnancy^45^.

Of course, in addition to the above factors, the occurrence and development of IRiP are also closely related to a history of polycystic ovary syndrome, environmental pollutants, sleep disorders during pregnancy, and mental and psychological factors.

## 3. Assessment methods of insulin resistance in pregnancy

Just as there are many methods for assessing IR in non-pregnant populations, there are also various approaches to evaluating IRiP. The world-recognized "gold standard" method mainly refers to the hyperinsulinemic euglycemic clamp (HEC) technique. Although these methods are more accurate, they are complex and invasive procedures, and thus are not suitable for pregnant women. In contrast, simpler indices constructed from parameters related to blood glucose and insulin are more applicable for large-scale scientific research or clinical studies, such as the homeostatic model assessment of insulin resistance (HOMA-IR), the quantitative insulin check index (QUICKI), and the Matsuda index, etc.

### 3.1 HOMA-IR

HOMA-IR is the most commonly used method to estimate IR based on fasting blood glucose and fasting insulin concentrations, which was first described by Matthews *et al*. in 1985^46^, ^47^. Currently, the formula proposed by Matthews and colleagues is widely recognized: [fasting blood glucose (mmol/L)×fasting insulin (µU/L)]/22.5^48^. The higher the HOMA-IR value, the stronger the IR level and the higher the risk of metabolic diseases^49^. In a retrospective cohort study of GDM patients in China, when HOMA-IR≥2.0, it was defined as high insulin resistance in pregnancy (high-IRiP). Compared with the low insulin resistance in pregnancy (low-IRiP) group, the high-IRiP group had a higher risk of large for gestational age infants (LGA) and gestational hypertension^45^. In another retrospective cohort study of GDM patients in China, GDM patients were divided into four groups from low to high based on HOMA-IR values, it was found that the higher the HOMA-IR value, the greater the risk of preterm birth^50^.

### 3.2 QUICKI

QUICKI is another index for evaluating IR, which was first proposed by A Katz *et al*. in 2000^51^. QUICKI can be calculated from fasting blood glucose and serum insulin samples^52^. The calculation formula is: 1/[log(fasting insulin)+log(fasting blood glucose)], QUICKI is significantly positively correlated with whole-body insulin sensitivity, the lower the QUICKI value, the stronger the IR level^53^. It also has the characteristics of simplicity, reliability, accuracy, and repeatability, it can not only assess IR but also predict the risk of type 2 diabetes to a certain extent^54^, ^55^. A prospective cohort study of Chinese women found that GDM patients with a high QUICKI index had a greater risk of LGA and excessive weight gain during pregnancy, but the risk of preterm birth decreased^56^.

### 3.3 Matsuda

The Matsuda index is another index for evaluating IR, which was first proposed by Matsuda and DeFronzo in 1999, its calculation formula is: 10000/[(fasting blood glucose×fasting insulin)×(average blood glucose concentration during OGTT (G_average_)×average insulin concentration during OGTT (I_average_))]^1/2^, where G_average_= (G_0_+G_30_+G_60_+G_120_+G_180_)/5 and I_average_= (I_0_+I_30_+I_60_+I_120_+I_180_ )/5, the lower the Matsuda index, the stronger the IR level. This index combines fasting and oral glucose tolerance test (OGTT) blood glucose and insulin data, comprehensively reflecting the insulin sensitivity of the liver and peripheral tissues, and is suitable for more precise IR assessment^57^. In a multicenter retrospective cohort study involving the United States, Northern Ireland, Thailand, Israel, Australia, Sweden, and China, high-IRiP was defined as the Matsuda index of GDM women being lower than the 25th percentile of normal glucose tolerance (NGT) women, it was found that compared with NGT women, the high-IRiP group had a higher risk of neonatal hypoglycemia, childhood obesity, and impaired glucose tolerance in children^58^.

## 4. Impact of insulin resistance in pregnancy on obstetric outcomes

When the degree of IRiP is significantly and inappropriately elevated, it can trigger pathological metabolic disorders, which may have adverse effects on both maternal and fetal health and obstetric outcomes, this includes inducing IR in fetal placental vascular endothelial cells, leading to a series of abnormal signal pathways and membrane transporter functions, and ultimately possibly causing placental vascular dysfunction, this dysfunction not only affects fetal growth and development during pregnancy but this is also closely related to the risk of long-term metabolic diseases in the offspring^39^. In addition, IR and hyperglycemia can lead to elevated levels of inflammatory markers such as C-reactive protein (CRP), plasminogen activator inhibitor-1 (PAI-1), and IL-6 in pregnant women, further exacerbating hyperinsulinemia, thereby altering vascular endothelial cell function and affecting placental vascular function in pregnant women^59^, ^60^. We have summarized some literature on the relationship between IRiP and obstetric outcomes in Table 2.

### 4.1 Impact on obstetric outcomes

A retrospective cohort study in China showed that among patients with GDM, the high-IRiP group had a higher risk of gestational hypertension and LGA compared with the low-IRiP group^45^. A similar finding was reported in a prospective cohort study of European-ancestry pregnant women in Canada, where even within the NGT group, the median z-score of neonatal birth weight was higher in the high-IRiP group than in the low-IRiP group^7^. In a prospective cohort study in Belgium, where compared with the NGT group, the high-IRiP group had a higher risk of preterm birth, neonatal hypoglycemia, NICU admission rate, induction of labor, and cesarean section rate, while the low-IRiP group had similar adverse obstetric outcomes to the NGT group. However, there was no statistical difference in obstetric outcomes between high-IRiP women and low-IRiP women. In this study, after adjusting for several variables such as BMI, fasting blood glucose, and lipid levels in early pregnancy, the risk of preterm birth and neonatal hypoglycemia in the high-IRiP group was still significantly higher than that in the NGT group, indicating that the increased risk of adverse obstetric outcomes in high-IRiP women cannot be fully explained by high levels of BMI, fasting blood glucose, and lipids^61^. Similar conclusions have also been reported in other literature^7^. Clearly, the above studies cannot reveal whether the effects of IRiP on the mother and fetus are independent of hyperglycemia in GDM.

All the above studies suggest that high-IRiP may have a negative impact on obstetric outcomes, but it is unclear whether this effect is due to the higher blood glucose levels in the high-IRiP group or the stronger IR and hyperinsulinemia in this group. A common shortcoming in previous literature is that the influence of this important confounding factor has not been analyzed. Interestingly, in another retrospective cohort study in China, after adjusting for confounding factors such as pre-pregnancy BMI and age, the risk of LGA in the GDM group with high-IRiP was similar to that in the NGT group, however, the risk of LGA in the GDM group with low-IRiP was significantly higher than that in the NGT group, this might be related to the fact that the GDM group with low-IRiP had a lower insulin secretion function and thus was less likely to achieve ideal blood glucose control. Unfortunately, this study did not compare the high-IRiP group and the low-IRiP group of pregnant women^62^. A multicenter, retrospective cohort study also showed that regardless of whether there was additional significant IR, the risk of LGA, neonatal hyperinsulinemia, and neonatal obesity in all groups of GDM was significantly higher than that in the NGT group, but the high-IRiP group had more adverse pregnancy outcomes in women^63^. Thus, the relationship between IR in pregnant women and obstetric outcomes is controversial, and some common deficiencies in related studies have affected the objective interpretation of the research results.

The reasons why women with IR have a higher risk of adverse pregnancy outcomes have not been fully clarified, but there are several seemingly reasonable biological explanations: firstly, IR is often accompanied by various metabolic changes, including elevated insulin levels and circulating lipids, elevated insulin levels in early pregnancy are associated with increased placental size and altered placental gene expression, while elevated plasma triglycerides are associated with neonatal obesity; secondly, women with higher IR levels have more difficulty controlling their blood sugar, so adverse obstetric outcomes may still be secondary to higher blood sugar levels^64^.

### 4.2 Long-term maternal and offspring outcomes after childbirth

Regarding the long-term effects of IRiP on mothers and infants after childbirth, a global multicenter retrospective cohort study revealed that the association between GDM in women and impaired glucose tolerance in their offspring seems to be mainly driven by the offspring of GDM mothers with IR as the main feature, compared with the NGT group of pregnant women, the offspring of GDM women with high-IRiP have an increased risk of obesity and impaired glucose tolerance in childhood, while there is no significant difference in the low-IRiP group. However, in this study, there was no direct comparison between women with high-IRiP and those with low-IRiP^58^. In a prospective cohort study in China, it was found that increased IR in patients with GDM is associated with an increased risk of early overweight in their offspring^65^. It can be seen that an inappropriate increase in IRiP in mothers can affect the insulin sensitivity of their offspring, which may have adverse effects on the early metabolism of their offspring after birth and may increase the risk of type 2 diabetes, obesity, and cardiovascular diseases in adulthood^66^.

Just as with adverse pregnancy outcomes, the impact of IRiP on postpartum glucose metabolism is also worthy of attention, but research in this area is scarce. A prospective cohort study from a group of women with GDM in Switzerland found that, especially among overweight or obese women with GDM, those women with high-IRiP had a significantly higher risk of developing postpartum impaired glucose tolerance compared to those with low-IRiP^67^. However, contrary to this conclusion, a prospective cohort study in Belgium showed that among women with GDM who underwent OGTT postpartum, there was no statistically significant difference in the risk of postpartum impaired glucose tolerance (impaired fasting glucose, impaired glucose tolerance, or both) between those with high-IRiP and those with low-IRiP^61^.

## 5. Intervention strategies of insulin resistance in pregnancy

In terms of diet, the DASH diet (Dietary Approaches to Stop Hypertension, which focuses on high-protein and high-fiber foods and limits saturated fat and added sugar in processed foods to lower blood pressure) has shown significant benefits in managing GDM, reducing postprandial and fasting blood glucose, lowering glycated hemoglobin, improving lipid profiles, and reducing systolic blood pressure, this dietary intervention has consistently been associated with better pregnancy outcomes, including a reduced rate of cesarean sections, lower birth weight, smaller head circumference, and a decreased incidence of macrosomia, combining the DASH diet with carbohydrate counting further improved glycemic control, significantly reduced IR, and decreased the proportion of patients with GDM requiring insulin treatment^68^.

In terms of exercise, from a public health perspective, active exercise before pregnancy may be a good strategy to reduce the risk of IR in the middle and late stages of pregnancy^69^. Regular exercise during pregnancy can improve metabolic disorders and mitochondrial quality, reduce inflammation and chronic oxidative stress caused by obesity and hyperglycemia, promote the adaptability of skeletal muscle, thereby enhancing oxidative capacity, increasing the expression of proteins involved in mitochondrial biogenesis, strengthening fat oxidation capacity, and improving insulin sensitivity and glucose uptake capacity^70^. Therefore, exercise can improve the IR level and overall health of pregnant women through multiple pathways, thereby exerting a positive impact on fetal metabolism.

In terms of medication, metformin is considered safe for use in the early stages of pregnancy as it does not increase the risk of major congenital malformations and its transfer from the mother to the fetus is minimal during this period^71^. Metformin can effectively reduce the incidence of GDM, enhance insulin sensitivity in women with GDM, and improve insulin response in women with gestational hyperglycemia by reducing postprandial blood glucose peaks^10^. However, metformin can easily cross the placenta, resulting in metformin levels in umbilical cord blood being as high or even higher than those in the mother, women with GDM who use metformin during pregnancy have offspring with a higher future BMI, waist circumference, and a higher risk of obesity^71^. Other drugs that can improve IR, such as glucagon-like peptide-1 (GLP-1) receptor agonists and thiazolidinediones, are not suitable for improving IRiP due to safety concerns during pregnancy.

In addition, some studies have also reported measures that have certain improvement effects on IRiP, such as supplementing inositol, vitamin D, probiotics, Omega-3, etc.^72^-^75^. Therefore, through changing diet, persisting in physical exercise and other ways, IRiP can be effectively improved.

## 6. Conclusion

This review discusses IRiP from several aspects, including its pathophysiology, influencing factors, assessment methods, and impacts on pregnancy outcomes. Variations in parameters used to assess IRiP, cut off values, gestational periods examined, and racial composition of study populations across different studies limit the generalizability of their findings. Since IRiP is closely associated with conditions such as pre-pregnancy and gestational obesity, hyperglycemia during pregnancy, and polycystic ovary syndrome, most current research on IRiP is linked to these comorbid states, leaving the significance of IRiP as an independent condition unclear. Most existing studies suggest that excessively high IRiP levels during pregnancy may increase the risk of adverse obstetric outcomes; however, it remains uncertain whether this effect stems from concomitantly elevated glucose levels or is independently mediated by hyperinsulinemia caused by high IRiP. To address this question, more refined subgroup analyses are needed within populations with similar glucose profiles during pregnancy. Furthermore, although some interventions targeting IRiP have been proposed, their long-term efficacy, safety, and sustainability require stronger evidence-based support. Future research could also expand to examine the dynamic trajectory of IRiP throughout pregnancy, which would offer a more integrated understanding of the evolving patterns of maternal glucose metabolism and placental function.

## Supplementary Material

Supplementary table.

## Figures and Tables

**Table 1:** SNPs related to IR in pregnant women

Gene	Function of the encoding protein	Location	The relationship with GDM
IRS1	A member of the IRS protein substrate family. An endogenous substrate of the insulin receptor and plays a crucial role in the insulin signaling pathway, being expressed in insulin-sensitive tissues^76^.	Located in the q36-37 region of the long arm of chromosome 2^77^.	In a study conducted in southern Sweden on women with a history of GDM, it was found that the C allele of IRS1 rs2943641 was associated with IR^78^.
MTNR1B	A melatonin MT2 receptor, which mediates the effects of melatonin on circadian rhythms, sleep and metabolism. Also known as melatonin receptor 1B. A member of the G protein-coupled receptor superfamily, who affects glucose tolerance and IR^79^, ^80^.	Located in the region of chromosome11, long arm, q14.3-q21^81^.	In a cross-sectional study conducted in multiple centers across four continents around the world, it was found that the MTNR1B rs10830963 genotype is associated with the risk of developing GDM mainly characterized by IRiP^80^.
GCKR	A glucose kinase regulatory protein, which is a protein that competitively inhibits liver glucose kinase^80^.	Located in the p23.3 region on the short arm of chromosome 2^82^.	In a cross-sectional study conducted in multiple centers across four continents around the world, it was indicated that the GCKR rs1260326 gene is associated with GDM characterized by IRiP^80^.
MIF	A crucial protein in the immune system and inflammatory responses^83^.	Located in the q11.2 region of the long arm of chromosome 2^83^.	In a study concerning the pregnant population in Iran, it was indicated that the MIF gene variation may affect IRiP and be related to GDM. Moreover, the MIF rs1007888gene polymorphism may influence gene expression, thereby affecting the occurrence of obesity and metabolic syndrome^83^.

GDM, Gestational Diabetes Mellitus; IRiP, Insulin Resistance in Pregnancy; IRS1, Insulin Receptor Substrate 1; MTNR1B, Melatonin Receptor 1B; GCKR, glucokinase regulator; MIF, Macrophage Migration Inhibitory Factor

**Table 2:** A literature summary on the relationship between IRiP and obstetric outcomes

Author	Year	Country	Method	Definition of high-IRiP in pregnant woman	Conclusion
Camille E.Powe *et al*.	2016	Canada	Prospective	The Matsuda index of GDM women is lower than the 25th percentile of NGT women	①Relative to women with NGT, women with high-IRiP had larger infant birth weights and greater risk of GDM associated adverse outcomes. ②Women with low-IRiP had infant birth weights, and risk of adverse outcomes similar to those in women with NGT.
Katrien Benhalima *et al*.	2019	Belgium	Prospective	The Matsuda index of GDM women is lower than the 50th percentile of NGT women	①Compared with women with NGT, women with high-IRiP had higher rates of preterm delivery, labour induction, caesarean section, neonatal hypoglycaemia and NICU admissions.②Women with low-IRiP had similar pregnancy outcomes as women with NGT.③The risk of postpartum glucose tolerance disorders (impaired fasting glucose, impaired glucose tolerance, or both) in women with high-IRiP was significantly higher than that in women with low-IRiP, but the difference was not statistically significant.
J.Immanuel *et al.*	2021	Nine European Countries	Retrospective	The HOMA-IR was above the median value in women with NGT	①Compared with women in the NGT group, women in the high-IRiP group had a greater risk of having LGA babies and caesarean section. ②Woman in the low-IRiP groups had similar pregnancy outcomes to those in the NGT group.
Meredith E.Osmulski *et al.*	2025	USA, North Ireland, Thailand, Israel, Australia, Sweden, China	Retrospective	The Matsuda index of GDM women is lower than the 25th percentile of NGT women	①Compared with women in the NGT group, women in the high-IRiP group had a greater risk of neonatal hypoglycemia, childhood obesity and a higher risk of child-impaired glucose tolerance.②Compared with women in the NGT group, the low-IRiP group had no significant correlation with adverse perinatal outcomes and adverse outcomes for the offspring in the future.
Yingfeng Liu *et al*.	2018	China	Prospective	The Matsuda index of GDM women is lower than the 25th percentile of NGT women	Compared with women with NGT, perinatal outcomes of women in the high-IRiP group exhibited no difference with women with NGT.
Ning WANG *et al*.	2021	China	Retrospective Case-Control	The Matsuda index of GDM women is lower than the 25th percentile of NGT women	①After adjusting for confounding factors such as pre-pregnancy BMI and age and so on, compared with women in the NGT group, the risk of LGA in the high-IRiP group was not significant, but in the low-IRiP group was still significant.②Compared with women in the NGT group, the risk of macrosomia in the high-IRiP group was not significant.
Lene R. Madsen *et al*.	2021	USA, China, Australia UK	Retrospective	The Matsuda index of GDM women is lower than the 25th percentile of NGT women	①Compared with women with NGT, the high-IRiP group had higher gestational weight gain and clearly demonstrated higher rates of adverse obstetric and neonatal outcomes, including primary caesarean delivery, LGA offspring, pregnancy-related hypertension, neonatal hyperinsulinaemia and neonatal adiposity.②Compared with women with NGT, women in the low-IRiP group also showed higher risks of LGA offspring, neonatal hyperinsulinaemia and neonatal adiposity.
Yixin Gong *et al*.	2024	China	Prospective	HOMA-IR, HOMA-IS, QUICKI-I, QUICKI-CP	①Compared with gestational-IGT, gestational-IFG had greater GDM-IR.②Compared with the NGT group, gestational-IFG was associated with a significantly higher risk of LGA; neonatal brain injury was significantly increased in the gestational-IGT group.③Gestational-IFG was more strongly associated with excessive gestational weight gain and LGA infants than gestational-IGT. Gestational-IGT showed greater risks of preterm birth compared to gestational-IFG.
Yiying Sun *et al*.	2020	China	Retrospective	HOMA-IR	Greater IR was associated with cesarean delivery, preterm delivery, macrosomia, and LGA offspring, but only significantly associated with preterm delivery after adjustment for potential confounders (age, pre-pregnancy BMI, weight gain before diagnosis of GDM).
Jing Lin *et al*.	2021	China	Retrospective	When HOMA-IR≥2.0, high-IRiP was diagnosed	Compared with women in the low-IRiP group, high-IRiP in the second trimester increased adverse pregnancy outcomes, especially the risk of hypertensive disorders of pregnancy and LGA.
Camille E.Powe *et al*.	2016	Canada	Prospective	The Matsuda index is lower than the 25th percentile of NGT women	In women with NGT, the median birth weight z score of infants born to mothers with high-IRiP was greater than in infants born to mothers with low-IRiP.
Anna Lesniara- Stachon *et al*.	2024	Switzerland	Prospective	Higher BMI, HOMA-IR, HOMA-β	Compared with the low-IRiP group, the high-IRiP group had a significantly higher risk of developing glucose intolerance one year after delivery.

IRiP, Insulin Resistance in Pregnancy; NGT, Normal Glucose Tolerance; GDM, Gestational Diabetes Mellitus; NICU, Neonatal Intensive Care Unit; LGA, Large for Gestational Age; IFG, Isolated Impaired Fasting Glucose; IGT, Isolated Impaired Postload Glucose Tolerance; HOMA-IR, Insulin Resistance index; HOMA-IS, Insulin sensitivity index; QUICKI-I, Quantitative insulin sensitivity check index calculated with insulin; QUICKI-CP, Quantitative insulin sensitivity check index calculated with C-peptide; BMI, Body Mass Index; HOMA-β, Insulin secretion index; The adjusted confounders in the data analysis of each study was shown in Table S1.
