# Supplementary material for: Progress and Practice of Insulin Resistance in Pregnancy: From Pathogenesis to Management
Source: Int J Med Sci. 2026 Sep 3;23(10):3252–61. doi: 10.7150/ijms.136420 (PMC13617488; doi:10.7150/ijms.136420)
Supplement: Supplementary file 1 [file ijmsv23p3252s1.pdf]

Supplements

Table S1 A literature summary on the relationship between IRiP and obstetric outcomes

Country and year	Method	Definition of high-IRiP in pregnant woman	Conclusion	Adjusted confounders
Canada, 2016	prospective	The Matsuda index of GDM women is lower than the 25th percentile of NGT women	①Relative to women with NGT, women with high-IRiP had larger infant birth weights and greater risk of GDM associated adverse outcomes.<br>②Women with low-IRiP had infant birth weights, and risk of adverse outcomes similar to those in women with NGT.	maternal BMI at OGTT
Belgium, 2019	prospective	The Matsuda index of GDM women is lower than the 50th percentile of NGT women	①Compared with women with NGT, women with high-IRiP had higher rates of preterm delivery, labour induction, caesarean section, neonatal hypoglycaemia and NICU admissions.<br>②Women with low-IRiP had similar pregnancy outcomes as women with NGT.<br>③The risk of postpartum glucose tolerance disorders (impaired fasting glucose, impaired glucose tolerance, or both) in women with high-IRiP was significantly higher than that in women with low-IRiP, but the difference was not statistically significant.	demographics, family history of diabetes, history of GDM and blood pressure, maternal BMI and waist circumference, fasting lipids, FPG and HbA1c in early pregnancy, and gestational weight gain until the OGTT at 26–28 weeks of pregnancy
Nine European Countries, 2021	retrospective	The HOMA-IR was above the median value in women with NGT	①Compared with women in the NGT group, women in the high-IRiP group had a greater risk of having LGA babies and caesarean section.<br>②Woman in the low-IRiP groups had similar pregnancy outcomes to those in the NGT group.	maternal age, pre-pregnancy BMI, ethnicity, country, maternal glycaemia (fasting, 1-h and 2-h glucose tolerance values, HbA1c) at OGTT, multiparity, family history of diabetes, smoking status, higher education, GDM treatment and DALI intervention
USA, North Ireland, Thailand, Israel, Australia, Sweden, China, 2025	retrospective	The Matsuda index of GDM women is lower than the 25th percentile of NGT women	①Compared with women in the NGT group, women in the high-IRiP group had a greater risk of neonatal hypoglycemia, childhood obesity and a higher risk of child-impaired glucose tolerance.<br>②Compared with women in the NGT group, the low-IRiP group had no significant correlation with adverse perinatal outcomes and adverse outcomes for the offspring in the future.	field center, age, height, mean arterial pressure, gestational age, parity, smoking, maternal BMI and alcohol consumption collected at OGTT

Continued Table S1   A literature summary on the relationship between IRiP and obstetric outcomes

Country and year	Method	Definition of high-IRiP in pregnant woman	Conclusion	Adjusted confounders
China, 2018	prospective	The Matsuda index of GDM women is lower than the 25th percentile of NGT women	Compared with women with NGT, perinatal outcomes of women in the high-IRiP group exhibited no difference with women with NGT.	age, pre-pregnancy BMI and maternal BMI at OGTT
China, 2021	retrospective case-control	The Matsuda index of GDM women is lower than the 25th percentile of NGT women	① After adjusting for confounding factors such as pre-pregnancy BMI and age and so on, compared with women in the NGT group, the risk of LGA in the high-IRiP group was not significant,but in the low-IRiP group was still significant.<br>②Compared with women in the NGT group, the risk of macrosomia in the high-IRiP group was not significant.	gestational weight gain, diet, or exercise during pregnancy and maternal age
USA, China, Australia UK, 2021	retrospective	The Matsuda index of GDM women is lower than the 25th percentile of NGT women	①Compared with women with NGT, the high-IRiP group had higher gestational weight gain and clearly demonstrated higher rates of adverse obstetric and neonatal outcomes, including primary caesarean delivery, LGA offspring, pregnancy-related hypertension, neonatal hyperinsulinaemia and neonatal adiposity.<br>②Compared with women with NGT, women in the low-IRiP group also showed higher risks of LGA offspring, neonatal hyperinsulinaemia and neonatal adiposity.	Oral Disposition Index z score and Matsuda z score, mean OGTT z score, maternal BMI at OGTT, ethnicity, age, height, smoking, parity, family history of diabetes, mean arterial blood pressure and gestational weight gain
China, 2024	prospective	HOMA-IR, HOMA-IS, QUICKI-I QUICKI-CP	① Compared with gestational-IGT, gestational-IFG had greater GDM-IR.<br>② Compared with the NGT group, gestational-IFG was associated with a significantly higher risk of LGA; neonatal brain injury was significantly increased in the gestational-IGT group.<br>③ Gestational-IFG was more strongly associated with excessive gestational weight gain and LGA infants than gestational-IGT. Gestational-IGT showed greater risks of preterm birth compared to gestational-IFG.	maternal age, education, and parity

Continued Table S1 A literature summary on the relationship between IRiP and obstetric outcomes

Country and year	Method	Definition of high-IRiP in pregnant woman	Conclusion	Adjusted confounders
China, 2020	retrospective	HOMA-IR	Greater IR was associated with cesarean delivery, preterm delivery, macrosomia, and LGA offspring, but only significantly associated with preterm delivery after adjustment for potential confounders (age, pre-pregnancy BMI, weight gain before diagnosis of GDM).	age, pre-pregnancy BMI, and weight gain before diagnosis of GDM
China, 2021	retrospective	When HOMA-IR≥2.0, high-IRiP was diagnosed	Compared with women in the low-IRiP group, high-IRiP in the second trimester increased adverse pregnancy outcomes, especially the risk of hypertensive disorders of pregnancy and LGA.	Age, parity, pre-pregnancy BMI , FPG, HbA1c, total cholesterol, triglycerides, total protein, albumin, HOMA-IR, gestational weight gain
Canada, 2016	prospective	The Matsuda index is lower than the 25th percentile of NGT women	In women with NGT, the median birth weight z score of infants born to mothers with high-IRiP was greater than in infants born to mothers with low-IRiP.	maternal BMI at OGTT
Switzerland, 2024	prospective	Higher BMI, HOMA-IR, HOMA-β	Compared with the low-IRiP group, the high-IRiP group had a significantly higher risk of developing glucose intolerance one year after delivery.	pre-pregnancy BMI, glucose 1-h during OGTT, FPG, previous history of GDM, HOMA-IR

IRiP, Insulin Resistance in Pregnancy; NGT, Normal Glucose Tolerance; GDM, Gestational Diabetes Mellitus; BMI, Body Mass Index; OGTT, Oral glucose tolerance test; NICU, Neonatal Intensive Care Unit; FPG, Fasting Plasma Glucose; HbA1c, Glycated Hemoglobin; LGA, Large for Gestational Age; DALI, Vitamin D And Lifestyle; IFG, Isolated Impaired Fasting Glucose; IGT, Isolated Impaired Postload Glucose Tolerance; HOMA-IR, Insulin Resistance index; HOMA-IS, Insulin sensitivity index; QUICKI-I, Quantitative insulin sensitivity check index calculated with insulin; QUICKI-CP, Quantitative insulin sensitivity check index calculated with C-peptide; HOMA-β, Insulin secretion index
